# Removal of Syndesmotic Screw After Fixation in Ankle Fractures: A Systematic Review

**DOI:** 10.7759/cureus.15435

**Published:** 2021-06-04

**Authors:** Omar Desouky, Amr Elseby, Ahmed H Ghalab

**Affiliations:** 1 Trauma and Orthopaedics, Royal Blackburn Hospital, Blackburn, GBR; 2 Family Medicine, Blackpool Victoria Hospital, Blackpool, GBR

**Keywords:** syndesmosis, ankle and foot, tibiofibular joint, ankle fracture, implant removal, functional outcomes, fixation of syndesmosis, retained screw

## Abstract

Syndesmotic injuries can occur with ankle fractures and can lead to destabilization of the ankle joint. As a result, it usually requires a transyndesmotic screw insertion to stabilize it. Currently, there is no consensus on the type, amount and diameter of screws used, the number of cortices needed to be engaged, the recommended time to weight-bearing, and whether the screw should be removed in these types of injuries. The aim of this study is to evaluate the evidence comparing the removal and non-removal of syndesmotic screws in open and closed ankle fractures that are associated with unstable syndesmosis in terms of functional, clinical, and radiological evidence. The study also looked at the evidence behind broken screw effects.

The literature search was conducted on March 16, 2021, using the Ovid Medline and Embase databases. The literature was eligible if it aimed to compare syndesmotic screw removal and retention in ankle fractures. One study found that those with a broken screw had a better clinical outcome than those with an intact screw. The studies were excluded if they were biomechanical studies, case reports, or were relevant but had no adequate English translation.

Initially, 53 studies were included but after scanning for eligibility, 11 were identified (including those added from references). Nine were cohort studies, seven of which did not find any difference in functional outcome between routine removal and retention of the syndesmotic screw. Two studies found there were better clinical outcomes in the broken screw group. Another study found that there were slightly worse functional outcomes in patients with intact screws as compared with those with broken, loosened, or removed screws. Two studies were randomized control studies that no significant functional outcomes between removed and intact syndesmotic screws. However, the majority of these studies had a high risk of bias.

Overall, the current literature provides no evidence to support routine removal of syndesmotic screws. Keeping in mind the clear complications and financial burden, syndesmotic screw removal should not be performed unless there is a clear indication. Furthermore, removal in the clinic, with the use of prophylactic antibiotics should be considered if indicated in cases with pain or loss of function. Further research in a structured randomized controlled trial (RCT) to examine if there is any difference in short- or long-term outcomes between removed, intact, loose, or broken syndesmotic screws might be beneficial. A multinational protocol for randomized control trials (RODEO-trial) is an example of such a study to determine the usefulness of on-demand and routine removal of screws.

## Introduction and background

Ankle fractures are one of the common presentations in the orthopaedics speciality. It is estimated that 75 per 100,000 adults who are <50 years old and 104 per 100,000 adults who are >50 years old presented with ankle fractures annually in the UK [1]. The British Orthopaedic Association has produced guidance on the management, as well as the follow-up, of ankle fracture [2]. It recommends the early fixation of unstable ankle fractures on radiological evidence, in ankle mortise view, in patients under 60 years old [2]. Syndesmotic injuries can occur with ankle fractures and can account for up to 20% of presentations [3]. Syndesmosis is considered to have healed between two and three months, and the screw is considered unnecessary [4]. Biomechanically, the evidence suggests that transyndesmotic fixation can limit ankle movement [5]. This is because the syndesmotic screw inhibits the physiological tibiofibular movement, which can affect dorsiflexion [5]. Currently, there is no consensus on the type, amount. and diameter of screws used, the number of cortices needed to be engaged, the recommended time to weight-bearing, and whether the screw should be removed in these types of injuries. Also, if the screws were to be removed, when is the best time to do so? Hence, the decision of syndesmotic screw removal is often left to expert opinion. Several studies have examined the routine removal of syndesmotic screws in the past; the majority of which showed no significant difference in outcome between retained or removed screws. Thus, the routine removal of syndesmotic screws was not recommended. However, they failed to critically review the radiological evidence and broken screws outcomes in correlation to these patients [6-7].

The aim of this systematic review is to examine the latest evidence comparing the removal and non-removal of syndesmotic screws in open and closed ankle fractures that are associated with unstable syndesmosis in terms of functional, clinical, and radiological evidence. This study will also examine the effects of broken screws for the same parameters.

## Review

Methods

The systematic review literature search was conducted on March 29, 2021. The Ovid Medline and Embase databases were searched using the following search terms: syndesmosis, syndesmotic, fixation of syndesmosis, syndesmotic screw, syndesmosis screw, transfixing screw, trans-syndesmotic screw, ankle Injuries, ankle fractures, bone screws, remov*, device removal, bone screw, syndesmosis screw removal, syndesmotic screw removal, removal of syndesmotic screw, removal of syndesmosis screw, screw removal, removal of screw, implant removal, hardware removal, screw, retention, retain*, in situ, intact, not removed, retained screw, retention of screw, retaining the screw, intact screw, comparative study. The entire search string could be viewed in the appendix. There was no limit to the year of publication or language used. A list was created based on the title and the abstract of the search. Studies that were found to not be relevant to the review were excluded at the initial stage of screening. The literature was then evaluated further and excluded if they were biomechanical studies, case reports, or were relevant but had no adequate English translation. The reference lists of those studies were reviewed, and relevant studies included. A study was eligible if it aimed to compare syndesmotic screw removal and retention in ankle fractures using patient recorded outcomes measures (PROMs) [8]. All the studies were reviewed by two authors for suitability and bias. The level of evidence of each study was evaluated using the Oxford criteria for level of evidence (OLOE) [9]. The quality of evidence of each study was reviewed by two authors independently using the Cochrane Risk of Bias 2 tool (RoB 2) [10] for randomized control trials (RCTs) and Methodological index for non-randomized studies (MINORS) [11] criteria for other types of comparative studies. The RoB 2 tool is a six-domain tool assessing bias through a trial’s designs, conduct, and reporting. Each domain is rated as either ‘Low’, ‘High’, or ‘Some concern’. The domain is deemed high risk of bias if a single rating within it is high risk, irrespective of other ratings. Otherwise, the domain is rated with the worst rating within it. MINORS is an instrument used to assess the quality of non-randomized studies. It has 12 main domains (with the last four dedicated for comparative studies). A score between 0 and 2 can be given to each domain, with 0 being not reported, 1 being not adequate, and 2 being adequate. Where mentioned in the studies, the patients with broken screws were classified as a separate group. Patients with loose screws were combined with the retained screw group of patients. The P-value of <0.05 was deemed statistically significant.

Results

**Figure 1:**
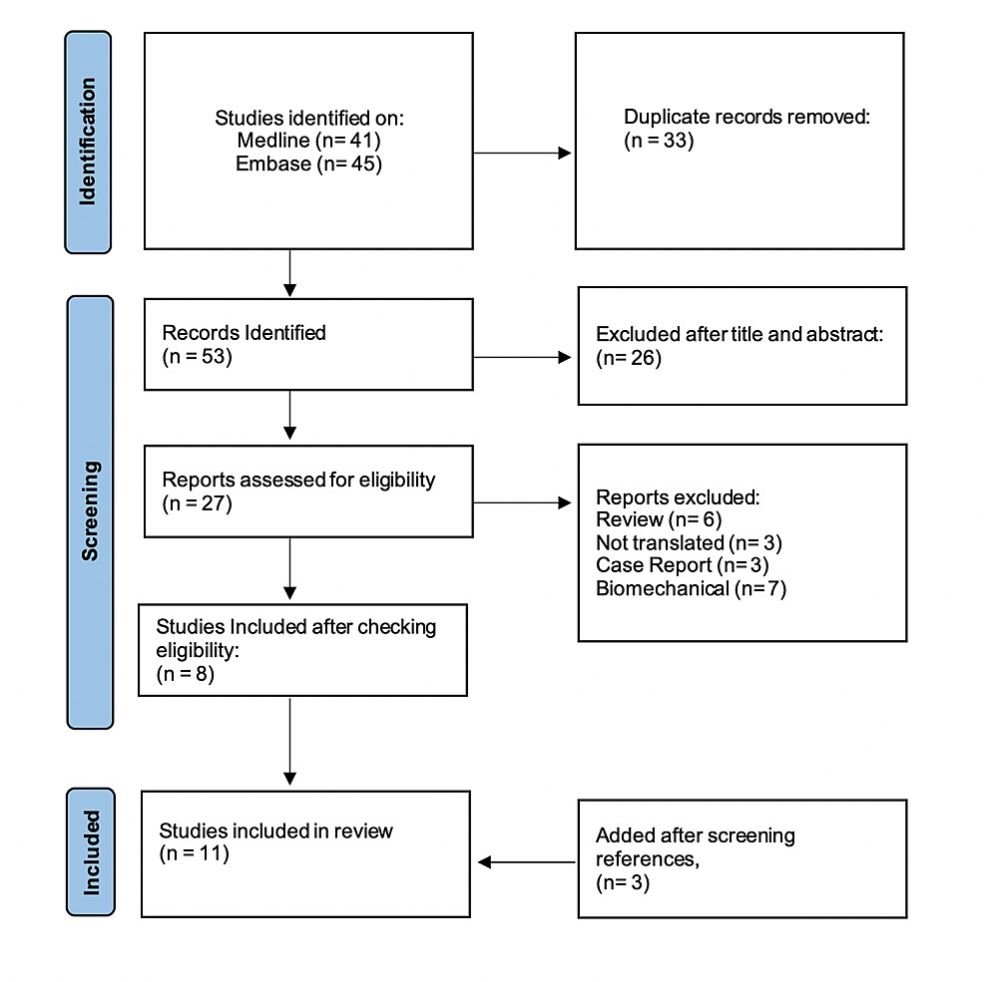
Flowchart including the literature search strategy as per the Preferred Reporting Items for Systematic Reviews and Meta-analysis (PRISMA) 2020 guidelines

Figure 1 shows the literature search process as per the Preferred Reporting Items for Systematic Reviews and Meta-analysis (PRISMA) 2020 guidelines [12]. A total of 11 studies describing outcomes if retained or removed screws were found. Those results were summarized in Table 1. Two of those studies were RCTs [13-14] and the rest were non-randomized cohort studies [15-23].

The majority of the studies compared elective removal versus retained screw (intact or broken). Functional and clinical outcomes were assessed using different scores, including Olerud-Molander ankle (OMAS) [24], American Orthopaedic Foot and Ankle Society (AOFAS) [25], American Academy of Orthopedic Surgeons foot and ankle score (AAOS), Baird and Jackson score [26], and Lower Extremity Measure (LEM). Patient satisfaction and pain score was determined by using the visual analogue scale (VAS) [27] in some studies.

In an RCT by Boyle et al. [14], they compared one-year post-operative outcomes following the retention or removal of syndesmotic screws in patients treated surgically for ankle fractures. Fifty-one (51) patients were randomly allocated to the retention of syndesmotic screw or removal at three months post-op. Outcomes measured were OMAS, AOFAS, AAOS, VAS, the mean active dorsiflexion and plantar flexion of the ankle, or the mean radiological tibiofibular clear space. The outcomes measured were to assess if there is any functional, clinical or radiological difference between the two groups. One year post-op, there was no significant difference in OMAS (84.2 retained vs 86.7 removed, p=0.367), there was also no significant difference found in all other outcomes measured.

In another RCT [13], they aimed to compare outcomes in 64 patients with ankle fractured, in which the syndesmosis was found to be unstable. Thirty (30) patients had fixation using one 4.5 mm cortical screw through both cortices, and 34 patients had fixation using two 3.5 mm cortical screws engaging only one cortex of the tibia. Two months after fixation, the quad cortical screws were routinely removed, whereas the tricortical screws were only removed if there was discomfort. The trial concluded that there was no significant difference in the functional score (OMAS 83.3 removed the quadcortical group vs OMAS 88.8 retained the tricortical group, p=0.192), pain, and dorsiflexion between the two groups after one year. The rest of the literature results are shown in Table 1.

**Table 1:** Current literature outcomes summary regarding syndesmotic screw removal OLOE: Oxford criteria for level of evidence; AOFAS: American Orthopaedic Foot and Ankle Society; OMAS: Olerud-Molander ankle score; VAS; visual analogue scale; AAOS: Orthopedic Surgeons foot and ankle score

Author	Type of study	Patients	OLOE	Mean follow-up	Left In (broken)	Removed	Time for removal	Outcome	Conclusion
Bell et al. 2006 [15]	Retrospective Cohort	33	4	15 months	7(2)	23	Removal after 6 to 12 weeks	BJS: Removed 88 vs Retained 86 (p=0.79)	No statistically significant difference between ankle scores. Incidence of screw breakage & osteolysis in retained group 6 months later.
Gennis et al. 2015 [16]	Retrospective Cohort	166	4	23 months	108(91)	58	12 weeks	Anteroposterior view Tibia-fibula clear space: Removed 4.1 vs Retained 4.0 P=0.762) Tibia-fibula Overlap: Removed 7.3 vs Retained 7.4 (P=0.76) Medial clear space: Removed 1.9 vs Retained 2.1 (P=0.541) Mortise view Tibia-fibula clear space: Removed 4.6 vs Retained 4.1 (P=0.024) Tibia-fibula Overlap: Removed 2.4 vs Retained 3.3 (P=0.033) Medial clear space: Removed 2.1 vs Retained 2.1 (P=0.839) *Final values in mm.	Removing the screw does not show a statistically significant difference in terms of the radiographic outcome of displacement of either the syndesmosis or mortise when compared to leaving it and whether it is intact or broken
Hsu et al. 2010 [17]	Retrospective Cohort	56	4	19 months	5(5)	47	G1 (n=19) = 6 weeks G2 (n=20) = 3 months G3 (n=13) = 9 months	Recurrence of syndesmotic diastasis G1, 3(15.8%). G2, 3(15%). G3, 0(0%). P=0.054 Breakage of syndesmotic screw G1, 0(0%). G2, 3(15%). G3, 2(15.4%) P=0.034 Satisfactory ankle function G1, 16(84.2%). G2, 16(80%). G3, 11(84.6%) P=0.191	Restricting daily activities for a minimum of 3 months is required to prevent syndesmotic diastasis. Removal of the screw at 6 weeks can prevent breakage but increases the likelihood of recurrence. Syndesmotic diastasis recurrence was not found to have any deterioration in ankle function over an average follow-up of 19 months.
Kaftandziev et al. 2015 [18]	Retrospective Cohort	82	4	12 months	59(13)	23	8-12 weeks	AOFAS (I=Intact, B=Broken, R= Removed) Group I: 83, Group B: 92.5, Group R: 85.5 (p=0.0496)	No statistical difference in clinical outcome found when comparing removed vs retained screw. However, the group with the broken screw had a statistically significant better clinical outcome when compared to the group with an intact screw.
Manjoo et al. 2010 [19]	Retrospective Cohort	106	4	23 months	51(not mentioned)	25	Mean 9 months	LEM (lower extremity measure) Intact screw 70±6 Broken, loosened or removed screws 85±3 (p=0.01) OMAS Intact screw 47±8 Broken, loosened or removed screws 64±4 (p=0.04)	Slightly worse functional outcomes in patients with intact screws compared with those with broken, loosened, or removed screws.
Schepers et al. 2014 [20]	Retrospective Cohort	122	4	51 months	12(0)	81	G1 (n=37) <8 week G2 (n=44) >8 weeks G3 (n= 12) retained	AOFAS G1 (94) vs G2 (90) vs G3 (92) OMAS G1 (82) vs G2 (73) vs G3 (73) VAS G1 (8.4) vs G2 (8.1) vs G3 (8.2)	No significant difference in clinical outcome between early, late and no removal. more stiffness reported by patients after 4.3 years in late and non-removal of syndesmotic screw groups.
Tucker et al. 2013 [21]	Retrospective Cohort	63	4	31 months	20	43	10-12 weeks	OMAS Removed 75 Retained 81.5 (p=0.107)	Retained-screw fixation does not substantially impair functional capacity, with additional cost-effectiveness, but when adjusted to gender (male) showed to be superior in retained as compared to removed
Hamid et al. 2009 [22]	Retrospective Cohort	52	4	30 months	37(10)	15	13 weeks	AOFAS Intact screw (n=27) 83.07 Broken screw (n=10) 92.40 Removed screw (n=15) 85.80 (p=0.0466)	Clinical outcomes did not differ in patients with intact or removed screws. Best clinical outcome in the broken group.
Moon et al. 2020 [23]	Retrospective Cohort	56	4	24 months	26(n/a)	26	(< 3 months pre-weight bearing)	AOFAS Group 1 (75.10±10.40) Group 2 (77.07±10.60) (p=0.487)	No statistical significance in clinical outcome between retained and removed, except in recurrent diastasis which was more in patients with the screw removed within 3 months.
Boyle et al. 2014 [14]	RCT	51	1b	12 months	25(9)	26	3 months	OMAS Retained 82.4 vs Removed 86.7 (p=0.367) AOFAS Retained 88.6 vs Removed 90.1 (p=0.688) AAOS Retained 96.3 vs Removed 94.0 (p=0.250)	Trans-syndesmotic screw removal yields no substantial functional, clinical, or radiological benefit in adult patients at 1-year follow up
Hoiness and Stromsoe 2004 [13]	RCT	64	2b	12 months	31 (3)	30	9.5 weeks	OMAS Quadcortical group removed 83.3; Tricortical group retained 88.8 (p=0.192)	No difference in functional outcomes between the two groups (removed a single quadcortical and retained two tricortical screws)

**Figure 2:**
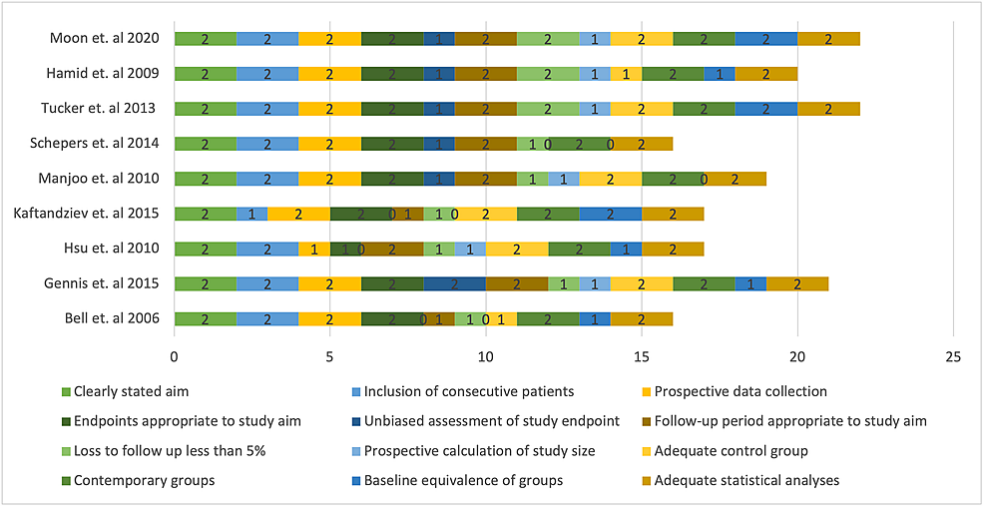
MINORS criteria risk of bias assessment MINORS: Methodological index for non-randomized studies

Figure 2 shows the risk bias assessment using MINORS criteria in nine cohort studies [11]. Table 2 shows the risk of bias according to the RoB 2 tool for the two RCTs studied in this literature [10].

**Table 2:** Cochrane’s RoB 2 tool RoB: risk of bias

Criteria	Boyle et al. [14]	Høiness and Strømsøe [13]
Randomization process	Low Risk	Low Risk
Deviation from Intended intervention	High Risk	High Risk
Missing outcome data	High Risk	High Risk
Measurement of outcome	High Risk	High Risk
Selection of reported results	Low Risk	Low Risk

Discussion

The literature review did not identify a significant difference in functional outcomes, suggesting routine removal of syndesmotic screws. This is supported by the two RCTs [13-14] that concluded that there were no significant functional outcomes between the removal or non-removal of screws.

The first randomized control study conducted by Boyle et al. [14] compared one-year post-operative outcomes following the retention or removal of syndesmotic screws (at three months) in patients treated surgically for ankle fractures. It concluded that the removal of a syndesmotic screw produced no significant functional, clinical, or radiological benefit in adult patients treated surgically for ankle fractures. In another RCT by Høiness & Strømsøe [13], there was no significant difference in functional score, pain, and dorsiflexion between either group. However, there is a concern regarding the methodology used in this study due to the use of different sizes and numbers of screws used in both groups, making it difficult to compare them. Manjoo et al. [19] found that there was a worse functional outcome associated with an intact screw as compared to loose, fractured, or removed screws. Furthermore, it highlighted that there might be a potential benefit in considering the removal of intact syndesmotic screws. However, the evidence was limited by its retrospective, non-randomized design, lack of a matched control population, and the small number of patients in each subgroup, which affects the power of the study.

There were two retrospective case studies looking at the necessity of syndesmotic screw removal prior to weight-bearing [15,23]. Both found that there was no statistically significant difference between ankle scores, functional outcomes, or range of motions between the removal and intact screw groups. However, the incidence of screw breakage and osteolysis were higher in the group where the screw was retained [15].

Kaftandzeiv et al. [18] and Hamid et al. [22] both found that the group with the broken screw had the best clinical outcome based on the AOFAS score when compared to the intact and removed screw groups. Kaftandzeiv et al. [18] found that the broken screw group had the best clinical outcome, which was statistically significant when compared to the intact screw group. However, this study was limited due to its retrospective design, short follow-up time, and low attendance rate. These findings were similar to Hsu et al. [17], who demonstrated that clinical outcomes were not negatively impacted when the syndesmotic screws were removed at six weeks, three months, or nine months. This study also noted a degree of tibiolofibular widening, which was also found by Hamid et al. [22]. However, this was not substantial enough to affect function. This was also corroborated by Gennis et al. [16], which found that the mortise remained intact and that there was no tibia-fibula diastasis after weight-bearing, whether the syndesmotic screws were removed or loosened or broken or remained intact. This evidence is also supported by Jordan et al. [28], which found that there was insignificant diastasis found upon removal of the screw, however, stable ankle mortise was achieved regardless.

Tucker et al. [21] concluded that the retained group achieved higher functional scores in each of the OMAS domains while also experiencing less pain when adjusted to the male gender. However, this study has several limitations, including the small population size and heavy reliance on the OMAS questionnaire, which is patient self-reported. Schepers et al. [20] found that there was no difference demonstrated in AOFAS, OMAS, or VAS scores in those who had their screws removed before eight weeks (minimum of six weeks), those who had it removed after eight weeks, or those who had it retained. However, it noted that patients with late removal or retained screws had higher rates of stiffness after 4.3 years. It also concluded that three cortical placement screws can behave more physiologically, thus removal was unnecessary in those group of patients as compared to using quadricortical and locking screws.

Removal of the screw has its associated risks and judging by the evidence produced by the literature, routine removal that is not clinically indicated cannot be recommended. Andersen et al. [29] found that there was a 5% wound infection rate after routine syndesmotic screw removal. The lack of pre-operative antibiotic prophylaxis, however, might have contributed to the infection rate. Schepers et al. [30] also found a high infection rate (9.2%) post syndesmotic removal, 2.6% of which were deep infection requiring re-operation. There was also recurrent syndesmotic diastasis in 6.6% of patients, and in 6.6%, screws were broken at the time of removal. However, as discussed previously, diastasis post screw removal or screw breakage seemed to affect functional or clinical outcome.

Tucker et al. [21] found that in view of cost implications and the lack of enough evidence to justify the routine removal of screw removal in the theatre, it was not recommended. Sugi et al. [31] analysed the safety and cost-effectiveness of syndesmotic screw removal in the clinic. One-hundred seventy (170) patients underwent the removal of syndesmotic screws with an overall infection rate of 1.2%. None of the patients who received prophylactic antibiotics (0 of 110) had an infection as compared to those who did not (2 out of 60). Cost savings of $13 829 per patient were achieved by syndesmotic screw removal in the clinic. This study had a lower infection rate (1.2%) compared to Andersen et al. and Schepers et al. (5% and 9.2%, respectively), implying that antibiotic prophylaxis might have an important role in reducing superficial wound infection post syndesmotic screw removal. The safety of removal with prophylactic antibiotics coupled with the cost-effectivity makes it a good alternative in indicated removals.

There are several limitations to the current study. Firstly, several studies fell into the criteria of the study, however, they were not included due to inadequate translation to the English language. This could lead to missing significant results, which could add to the quality of evidence in the literature. Also, it was difficult to perform a meta-analysis due to the heterogenicity of the results of the studies. A more structured RCT would be required to carry out a full meta-analysis to determine the significance of these findings. Such an RCT protocol was designed by Dingmans et al. in 2018 [32] with a robust methodology, however, it has yet to be carried out.

## Conclusions

The current literature provides no evidence to support the routine removal of syndesmotic screws. Keeping in mind the clear complications and financial burden, syndesmotic screw removal should not be performed unless there is a clear indication. Furthermore, removal in the clinic with the use of prophylactic antibiotics should be considered if indicated in cases with pain or loss of function. Further research in a structured RCT, to examine if there is any difference in short- or long-term outcomes between removed, intact, loose, or broken syndesmotic screws, might be beneficial. A multinational protocol for randomized control trials (RODEO-trial) is an example of such a study to determine the usefulness of on-demand and routine removal of screws.

## Appendices

Search strategy

Database: Ovid MEDLINE(R) ALL <1946 to March 29, 2021> Search Strategy: -------------------------------------------------------------------------------- 1 (Syndesmosis or syndesmotic).mp. [mp=title, abstract, original title, name of substance word, subject heading word, floating sub-heading word, keyword heading word, organism supplementary concept word, protocol supplementary concept word, rare disease supplementary concept word, unique identifier, synonyms] (1564)

2 "fixation of syndesmosis".mp. [mp=title, abstract, original title, name of substance word, subject heading word, floating sub-heading word, keyword heading word, organism supplementary concept word, protocol supplementary concept word, rare disease supplementary concept word, unique identifier, synonyms] (20)

3 ("syndesmotic screw" or "syndesmosis screw").mp. [mp=title, abstract, original title, name of substance word, subject heading word, floating sub-heading word, keyword heading word, organism supplementary concept word, protocol supplementary concept word, rare disease supplementary concept word, unique identifier, synonyms] (231)

4 ("transfixing screw" or "trans-syndesmotic screw").mp. [mp=title, abstract, original title, name of substance word, subject heading word, floating sub-heading word, keyword heading word, organism supplementary concept word, protocol supplementary concept word, rare disease supplementary concept word, unique identifier, synonyms] (18)

5  Ankle Injuries/ or Ankle Fractures/ (11432)

6  Bone Screws/ (23616)

7  remov*.mp. [mp=title, abstract, original title, name of substance word, subject

heading word, floating sub-heading word, keyword heading word, organism supplementary concept word, protocol supplementary concept word, rare disease supplementary concept word, unique identifier, synonyms] (676526)

8  Device Removal/ (13821)

9  ("syndesmosis screw removal" or "syndesmotic screw removal" or "removal of

syndesmotic screw" or "removal of syndesmosis screw").mp. [mp=title, abstract, original title, name of substance word, subject heading word, floating sub-heading word, keyword heading word, organism supplementary concept word, protocol supplementary concept word, rare disease supplementary concept word, unique identifier, synonyms] (31) 10 ("screw removal" or "removal of screw").mp. [mp=title, abstract, original title, name of substance word, subject heading word, floating sub-heading word, keyword heading word, organism supplementary concept word, protocol supplementary concept word, rare disease supplementary concept word, unique identifier, synonyms] (482) 11 ("implant removal" or "device removal").mp. [mp=title, abstract, original title, name of substance word, subject heading word, floating sub-heading word, keyword heading word, organism supplementary concept word, protocol supplementary concept word, rare disease supplementary concept word, unique identifier, synonyms] (16565) 12 "hardware removal".mp. [mp=title, abstract, original title, name of substance word, subject heading word, floating sub-heading word, keyword heading word, organism supplementary concept word, protocol supplementary concept word, rare disease supplementary concept word, unique identifier, synonyms] (1129) 13 screw.mp. [mp=title, abstract, original title, name of substance word, subject heading word, floating sub-heading word, keyword heading word, organism supplementary concept word, protocol supplementary concept word, rare disease supplementary concept word, unique identifier, synonyms] (35297) 14 (retention or retain* or "in situ" or intact or "not removed").mp. [mp=title, abstract, original title, name of substance word, subject heading word, floating sub- heading word, keyword heading word, organism supplementary concept word, protocol supplementary concept word, rare disease supplementary concept word, unique identifier, synonyms] (988905) 15 ("retained screw" or "retention of screw" or "retaining the screw" or "intact

screw").mp. [mp=title, abstract, original title, name of substance word, subject heading word, floating sub-heading word, keyword heading word, organism supplementary concept word, protocol supplementary concept word, rare disease supplementary concept word, unique identifier, synonyms] (97)

16  "comparative study".mp. or Comparative Study/ (1921457)

17  1 or 5 (12018)

18  6 or 13 (44871)

19  7 or 8 (676526)

20  14 and 17 and 18 and 19 (37)

21  3 or 4 (234)

22  14 and 19 and 21 (24)

23  8 or 9 or 10 or 11 or 12 (17781)

24  14 or 15 (988905)

25  17 and 23 and 24 (31)

26  20 or 22 or 25 (41)

Database: Embase <1974 to March 29, 2021> Search Strategy: -------------------------------------------------------------------------------- 1 (Syndesmosis or syndesmotic).mp. [mp=title, abstract, heading word, drug trade name, original title, device manufacturer, drug manufacturer, device trade name, keyword, floating subheading word, candidate term word] (1849)

2 ("syndesmotic screw" or "syndesmosis screw").mp. [mp=title, abstract, heading word, drug trade name, original title, device manufacturer, drug manufacturer, device trade name, keyword, floating subheading word, candidate term word] (288) 3 ("transfixing screw" or "trans-syndesmotic screw").mp. [mp=title, abstract, heading word, drug trade name, original title, device manufacturer, drug manufacturer, device trade name, keyword, floating subheading word, candidate term word] (19)

4  ankle injury/ (7235)

5  bone screw/ (25273)

6  remov*.mp. [mp=title, abstract, heading word, drug trade name, original title,

device manufacturer, drug manufacturer, device trade name, keyword, floating subheading word, candidate term word] (862900)

7  "Device Removal".mp. or device removal/ (21665)

8  ("syndesmosis screw removal" or "syndesmotic screw removal" or "removal of

syndesmotic screw" or "removal of syndesmosis screw").mp. [mp=title, abstract, heading word, drug trade name, original title, device manufacturer, drug manufacturer, device trade name, keyword, floating subheading word, candidate term word] (32) 9 ("screw removal" or "removal of screw").mp. [mp=title, abstract, heading word, drug trade name, original title, device manufacturer, drug manufacturer, device trade name, keyword, floating subheading word, candidate term word] (550) 10 ("implant removal" or "hardware removal").mp. [mp=title, abstract, heading word, drug trade name, original title, device manufacturer, drug manufacturer, device trade name, keyword, floating subheading word, candidate term word] (4104) 11 screw.mp. [mp=title, abstract, heading word, drug trade name, original title, device manufacturer, drug manufacturer, device trade name, keyword, floating subheading word, candidate term word] (59318) 12 (retention or retain* or "in situ" or intact or "not removed").mp. [mp=title, abstract, heading word, drug trade name, original title, device manufacturer, drug manufacturer, device trade name, keyword, floating subheading word, candidate term word] (1193535) 13 ("retained screw" or "retention of screw" or "retaining the screw" or "intact screw").mp. [mp=title, abstract, heading word, drug trade name, original title, device manufacturer, drug manufacturer, device trade name, keyword, floating subheading word, candidate term word] (103)

14  1 or 4 (8586)

15  5 or 11 (59318)

16  6 or 7 or 8 or 9 or 10 (862900)

17  12 or 13 (1193535)

18  14 and 15 and 16 and 17 (45)
